# Reverse Genetic Strategies in *Caenorhabditis elegans*: Towards Controlled Manipulation of the Genome

**DOI:** 10.1100/tsw.2011.126

**Published:** 2010-07-07

**Authors:** Howard A. Baylis, Rafael P. Vázquez-Manrique

**Affiliations:** ^1^ Department of Zoology, University of Cambridge, Cambridge, UK; ^2^ Laboratory of Neuronal Cell Biology and Pathology, Psychiatry and Neurosciences Centre INSERM U894, Paris, France

**Keywords:** biolistic transformation, targeted gene knock-out, gene engineering, mutagenesis, homologous recombination, Flp recombinase, transposable element, zinc-finger nuclease, Tc1, Mos1, MosTIC, MosDEL, plc-4, phospholipase C-delta

## Abstract

*Caenorhabditis elegans* has a complete annotated genome sequence that is augmented by increasing quantities of data from high-throughput postgenomic analyses. This has led to an increasing need to identify the biological functions of specific genes using reverse genetics, i.e., moving from gene to phenotype. Fundamental to this aim is the ability to alter the structure of particular genes by means that are not accessible to classical genetic strategies. Thus, one dream of *C. elegans* researchers is to establish a toolkit for the controlled manipulation of any *loci* within the genome. Although *C. elegans* is amenable to a wide variety of genetic and molecular manipulations, controlled manipulation of endogenous genes by, for example, gene targeting has proved elusive until relatively recently. In this review, we describe and discuss the different methods available for the inactivation and modification of endogenous loci with a focus on strategies that permit some measure of control in this process. We describe methods that use random mutagenesis to isolate mutations in specific genes. We then focus on techniques that allow controlled manipulation of the genome: gene modification by transposon mobilisation, gene knock-out mediated by zinc-finger nucleases, and gene targeting by biolistic transformation.

## FROM FORWARD TO REVERSE GENETIC STRATEGIES

*Caenorhabditis elegans* was initially established as a system in which to address fundamental questions about animal development and neurobiology using the power of genetic analysis. Since then, it has become a model system in which a wide variety of biological questions have been addressed. To investigate such questions, "forward genetic" strategies have been widely and very successfully adopted. In such approaches, mutants with a particular phenotype are identified, the underlying mutation is mapped genetically, and the relevant gene is cloned and characterised. To produce mutations, worms may be exposed to chemicals or ionising radiation that cause mutagenic changes in their DNA. Alternatively, mutations may be induced by the mobilisation of transposable elements. This type of forward genetic strategy is still widely used by biologists and has recently been augmented by the advent of rapid, whole-genome sequencing and deep sequencing strategies[1]. However, ever since the sequencing and annotation of *C. elegans,* genome reverse genetics, i.e., moving from gene to phenotype(s), has been chosen by many researchers to investigate the function of particular genes of interest. Such approaches make it possible to study, for example, the *C. elegans* homologues of genes involved in human disease or to establish *in vivo* roles for proteins with well-defined biochemical functions.

To perform reverse genetics, it is necessary to be able to manipulate the structure and/or expression of the gene of interest. In this review, we discuss the various strategies that have been adopted to do this, with particular emphasis on those that promise controlled manipulation of specific genes within the genome.

At its most basic, reverse genetics focuses on the ability to knock out or knock down the function of a specific gene. Both stable and transient approaches have been used in order to achieve this goal. Transient approaches offer many advantages in terms of speed and ease of use. Notably, RNA interference (RNAi) is particularly straightforward in *C. elegans*, requiring only the successful delivery of double-stranded RNA (dsRNA). Delivery of dsRNA for the target gene can be achieved by injection[2], by soaking animals in dsRNA[3], or by simply feeding animals with bacteria expressing the appropriate dsRNA[3]. RNAi has been used to target the orthologues of many genes involved in human disorders[4,5,6,7,8,9,10]. Importantly, RNAi and the ease with which it can be induced in *C. elegans* provided a breakthrough that made it possible to perform wide-scale screens in which large numbers of genes are targeted (Table 1). Such screens, as well as the more routine use of smaller-scale RNAi experiments, are greatly facilitated by the availability of libraries of bacterial clones expressing dsRNA for most of the genes of the *C. elegans* genome[11,12]. Further, such experiments are relatively low cost. A second transient approach, which is used in other organisms, is gene knock-down using morpholinos. Whilst this approach has not been exploited in *C. elegans,* it has been used in another model nematode, *Pristionchus pacificus*[13,14], and may yet prove to be useful in *C. elegans* when RNAi is not effective. One issue associated with RNAi is that the level of knock-down is variable and complete ablation of gene expression is probably rare. This, in turn, means that the researcher may observe a partial loss-of-function phenotype or even no phenotype at all. It may therefore be desirable to obtain structural mutants that produce a clean removal of the gene function. A number of approaches to doing this have been adopted and are discussed below. They can be broadly grouped into two classes: (1) those based on hunting for desired mutations in populations of randomly mutagenised animals and (2) those based on inducing specific changes in the gene of interest. We begin by discussing the first type.

## Generating Mutations in Specific Genes using Random Mutagenesis

One successful approach to generating deletion mutants in genes of interest is based on the generation of random deletions in the genome followed by PCR to identify those mutations that are located within the appropriate gene[15]. A number of versions of this general approach have been adopted (see, for example, [16,17,18]). A range of mutagens, e.g., EMS (ethyl methanesulfonate) and UV/psoralen, have been employed and a variety of approaches to the division, sib selection, and maintenance of the resulting populations of mutagenised worms have been used. Lysates from mutagenised populations are screened using primers that flank the target gene, thus allowing the identification of worms carrying substantial changes in the gene's DNA, usually relatively large deletions. This approach has been used, with substantial success, by two international programs with the aim of obtaining mutants of every gene in the worm: The *C. elegans* Gene Knockout Consortium (http://celeganskoconsortium.omrf.org/), and the National Bioresource Project for the Experimental Animal "Nematode *C. elegans*" (http://www.shigen.nig.ac.jp/c.elegans/). These two programmes have already obtained several thousands of mutants (Table 1)[19,20]. Random chemical mutagenesis can also be used to isolate point mutations in a gene of interest. TILLING (Targeting Induced Local Lesions in Genomes) was originally developed to isolate

**TABLE 1**
**Genes Targeted by Different Methods of Reverse Genetics in *C. elegans***

| Method | Successfully Targeted Genes | Ref. |
|---|---|---|
| RNAi | Whole genome several times<br>Large subsets of genes<br>Gene families<br>Many individual genes | [11,87,88,89,90, 91,92,93,94] |
| Chemical mutagenesis | Thousands | [95] |
| Gene conversion using transposons | Several targets | [40,42,45] |
| Transposon-directed mutagenesis | Several targets | [27,32,96,97] |
| Zinc-finger nucleases | One noncoding target | [56] |
| Gene targeting by homologous recombination and injection | *vit-2* | [68] |
| Gene targeting by homologous recombination and biolistic transformation | *unc-22* and *unc-54* | [70] |
| | *zhp-3* | [74] |
| | plc-4 | [75] |

mutant alleles in plants[21,22]. The technique relies on PCR amplification, using labelled primers, of the target DNA from mutagenised and nonmutagenised strains. DNA from the two strains is annealed and then digested with one of a number of enzymes that cleave at mismatched positions in the duplex. The products of the digestion are then resolved to identify mutant alleles. Gilchrist et al. used this strategy to isolate 71 mutant alleles in 10 selected genes from *C. elegans*[23], clearly demonstrating the power of this approach.

A second method of generating random mutations is through the mobilisation of transposable elements. Both endogenous transposable elements, such as *Tc1*, and exogenous elements, such as *Mos1*, have been used in *C. elegans*. In both cases, the defined sequence of the transposable element offers advantages in both gene identification and PCR screening for mutants. Earlier approaches utilised the mobilisation of *Tc1*, which is naturally present in all strains of *C. elegans*[24]. Insertion of *Tc1* may result in an altered gene product by itself, although this is rare, as *Tc1* tends to insert in introns and even when located in exons is often removed from the mRNA during RNA splicing[25]. Much more useful is the fact that mobilisation of a *Tc1* insertion may generate deletions due to defective repair of double strand breaks created during transposition. Thus, having identified a transposon in, or near to, a gene of interest, it is then possible to generate deletions in that gene by mobilising the transposon. Such strategies therefore depend on establishing populations that have a variety of random transposon insertions and then identifying useful transposon insertions by screening large numbers of worms by PCR. This approach was greatly facilitated by the use of frozen libraries of worms, in which *Tc1* had been mobilised[26,27]. Due to the low rate of transposition of *Tc1*, in the standard wild-type strain (Bristol N2), *mutator* backgrounds that promote higher rates of mobilisation, like the Bergerac strain[28], are used. This has been a useful strategy and a number of genes have been targeted using this technique (Table 1), although it has now been largely superseded by the use of the chemical mutagens described above. The use of *Tc1* poses some problems. First, genomes contain several copies of *Tc1* elements and, hence, their mobilisation from unwanted *loci* may lead to undesirable secondary mutations[29]. Second, the use of *mutator* backgrounds may produce the mobilisation of other transposable elements of the *Tc1*/*mariner* family also present in *C. elegans*[30].

To circumvent some of the problems associated with the use of an endogenous transposable element, *Mos1*, a transposon of the *Tc1*/*mariner* family from *Drosophila*[31], has been used. Bessereau and coworkers showed that this transposable element is active in the germ line of *C. elegans*[32] and that the only requirement for these elements to be mobilised is the *Mos1* transposase[32]. *Mos1* insertions are often mutagenic in themselves. Importantly, expression of the *Mos1* transposase does not induce mobilisation of endogenous transposons in *C. elegans*[32]. Further, the transposase can be provided transiently, via a transgene, so that once it is removed, the insertion of interest will be stable. This technique has been used to obtain mutant alleles in several different genes (Table 1). It has also provided a useful tool in forward genetic strategies. Projects to characterise large numbers of both *Tc1* and *Mos1* insertion sites in isolated worm strains have led to the production of databases that contain the exact location of large numbers of transposable element insertions sites (NemaGENETAG project (http://elegans.imbb.forth.gr/nemagenetag/)[33,34] (reviewed by Moerman and Barstead[20]).

The strategies described above have proven utility, are readily usable, and lend themselves to high-throughput screening. However, they do have some disadvantages. The use of chemical mutagens or transposable elements, as with all such mutagenesis-based strategies, results in strains that carry a range of other mutations that need to be taken into consideration and/or removed. In this regard, results from whole-genome sequencing have confirmed that mutant strains, obtained by EMS mutagenesis, possess a high load of "secondary" mutations, even after outcrossing[35]. Incidentally, the positive side of this background is that mutagenised genomes offer a potentially enormous library of useful mutations once these are identified by whole-genome sequencing and collated.

The initial investment in establishing libraries of mutagenised worms can be substantial and isolation of the desired targeted gene requires laborious molecular screening. Finally, the random nature of deletions generated means that in addition to putative loss-of-function mutations, deletions with less predictable effects on gene function may also be isolated. Whilst this could be advantageous if a range of deletions were available, in practice, only one or two deletions in any given gene are usually isolated. However, none of this detracts from the enormous impact and success associated with the application of these methods.

## METHODS FOR CONTROLLED GENE TARGETING

We now turn to attempts to introduce specific changes in the genome that overcome some of the disadvantages in the approaches described above. We will examine gene conversion by transposable elements, gene targeting mediated by zinc-finger nucleases, and gene-targeting methods based on biolistic bombardment. All these strategies reduce the risk of producing genomic lesions outside of the target and, more importantly, allow clean and controlled introduction of modifications in the genome of *C. elegans*. Such modifications may go beyond deletions to include the insertion of specific DNA fragments, such as tags or markers, or site-specific changes to produce more subtly modified gene products or conditional gene knock-outs.

### Transposon-Induced Genome Manipulation

DNA in cells is constantly being damaged by endogenous or environmentally induced events. Hence, cells possess machinery to repair such lesions[36,37]. Double-stranded DNA breaks (DSB) are among the most deleterious genomic lesions since they may cause genomic instability (see Kass and Jasin[38] for an update). Most DSB are repaired in cells through one of two pathways[36,37,38]. The first of these, nonhomologous end joining (NHEJ), may induce small deletions or insertions (Fig. 1). The second employs homologous recombination to repair the lesion using a homologous strand of DNA, which may be provided by the homologous chromosome or, importantly, from exogenous DNA (Fig. 1). Hence, DSB are strong inducers of homologous recombination[36,37,38]. In this regard, it was proposed that DSB produced by transposon mobilisation are repaired using homologous sequences in *C. elegans*[39]. This suggestion was based on experiments performed using strains that possess a *Tc1* transposon insertion within the *unc-22* locus[40]. The authors showed that the frequency of loss of *Tc1* insertions in this gene was

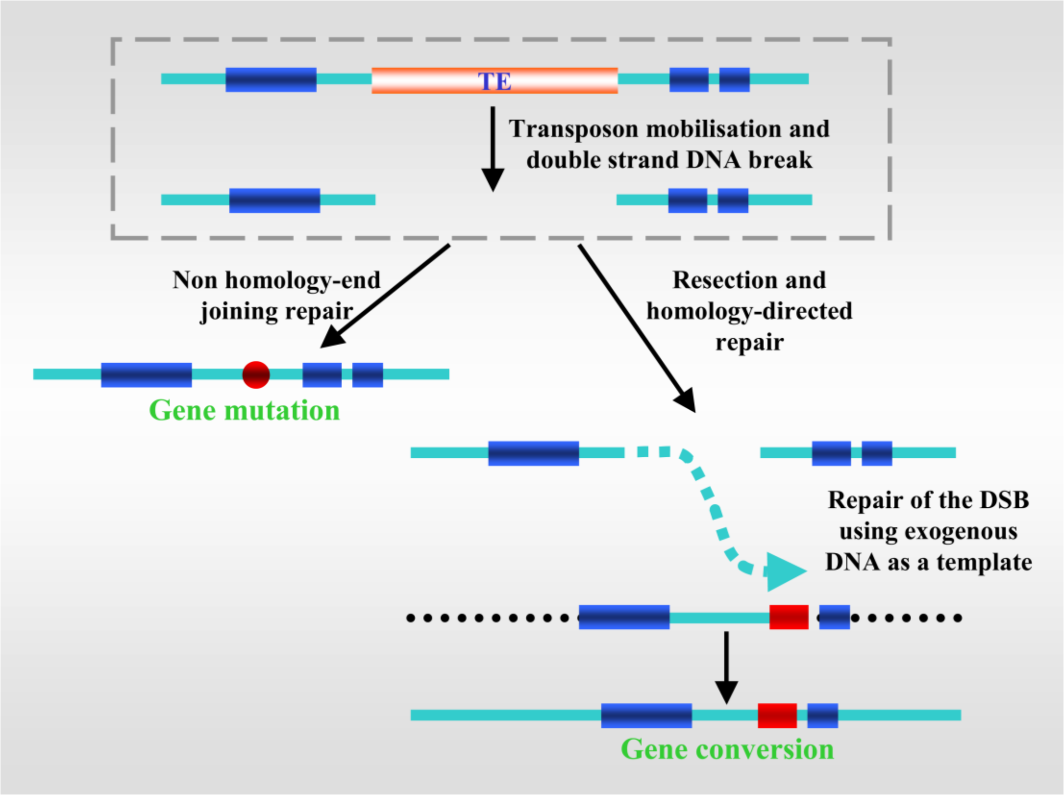

**FIGURE 1.** Mutagenesis and gene conversion driven by transposable element mobilisation. After mobilisation of a transposable element, a DSB is created. The presence of the DSB activates the endogenous repair machinery in the cell. The lesion may then be removed by direct end-joining repair (left arrow). This type of repair may result in the introduction of small insertions or deletions (represented in the diagram by a red dot). If, in contrast, the free DNA ends are resected, the resulting gap may then be repaired using a homologous strand of DNA (right arrow). The homologous template may be a homologous chromosome resulting in gene repair or an exogenous sequence, with partial homology to the damaged chromatid, which will result in gene conversion (represented in the diagram by a red bar). Double DNA strands are shown in blue. Exogenous DNA is shown by a dotted black line.

strongly influenced by the nature of the homologous DNA present[40]. *Tc1* losses in homozygous strains were rare, whereas losses in heterozygous animals were 100 times more frequent[40]. These experiments led the authors to hypothesise that the mobilisation of the transposable elements produced a DSB and that this was repaired using the homologous chromosome as a template. Hence, in homozygous animals, the lesion would be repaired using the homologous chromosome containing a copy of *Tc1* so that the transposon would not be lost, whilst in heterozygous animals, the transposon will be lost. Similar conclusions were reached after experiments on the mobilisation of the *Drosophila* P element[41]. Plasterk and Groenen took advantage of these naturally occurring events to engineer changes in the *unc-22* gene (Fig. 2)[40]. They used transgenes that contained polymorphisms that were not present in the genome of the *unc-22* strain, as donor DNA[40]. After mobilisation of the transposable element, they observed that sequences from the transgene were present in the genome of these animals[40]. However, the number of such events was low[40] and this potentially powerful technique for gene conversion was not exploited until improvements were introduced by Barrett and coworkers[42]. These authors targeted two *loci*, *trk-1* and *frm-3*, inducing substantial changes in the target, such as deletions and insertions, which importantly included the insertion of an in-frame GFP sequence in *frm-3*, thus showing the potential for engineering changes

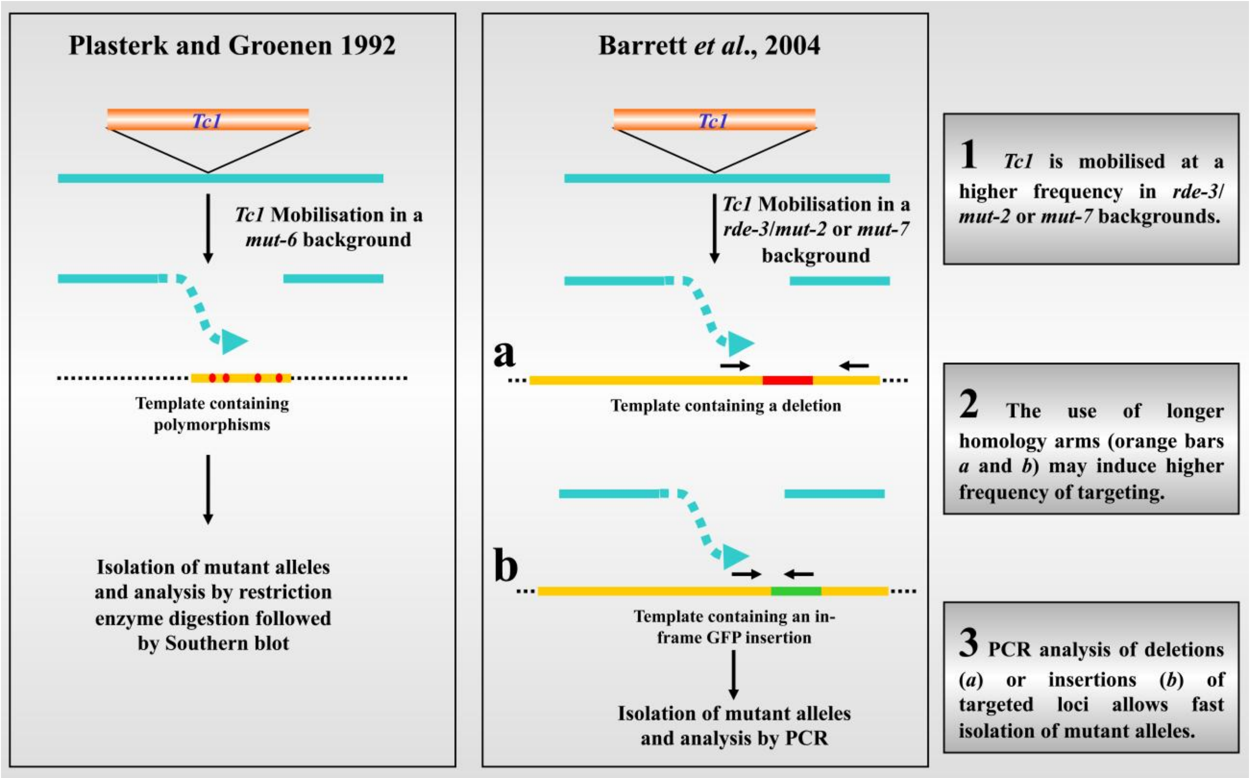

**FIGURE 2.** Diagram showing the improvements introduced by Barrett and coworkers to the method of gene conversion by Plasterk and Groenen. The first box shows a diagram that represents the technique developed by Plasterk and Groenen[40]. The second box represents the method by Barrett and coworkers[42]. The text boxes on the right-hand side highlight the three key improvements that made the method of Barrett and coworkers more effective. Blue bars represent genomic DNA. Orange bars represent exogenous DNA, homologous to the region to be targeted, used as a template to introduce changes in the genome. Arrows represent primers used for PCR analysis. Dotted black lines represent nonhomologous DNA.

in the genome by gene conversion[42]. Barrett et al. achieved increased efficiency of targeting events compared to those recorded by Plasterk and Groenen[40], which may have resulted from three improvements (Fig. 2)[42]. First, they screened for targeting events by PCR, on pooled populations of treated worms, using primers that only amplified engineered alleles (Fig. 2) [42]. Second, mutants of the RNAi pathway *rde-3*/*mut-2* or *mut-7* (Fig. 2), rather than the *mutator* strain *mut-6*, were utilised[30,43]. Finally, Barrett et al. used longer homology arms in the targeting constructs (Fig. 2), which may well have increased the probability of homologous recombination events.

The use of *Tc1* to induce DSB to promote homologous recombination gave the *C. elegans* community a powerful tool to manipulate the genome. However, as discussed above, the use of endogenous transposable elements may lead to instability of other copies of *Tc1* and similar transposons. Once again, the exogenous *Mos1* transposon has been used to circumvent this problem. Boulin and Bessereau[44] and Robert and Bessereau[45] analysed DSB repair after mobilisation of *Mos1* and showed that by using transgenes as donor DNA, it was possible to engineer controlled changes in the *C. elegans* genome; the so-called *Mos*TIC technology (*Mos1* excision-induced transgene instructed gene conversion)[44,45]. Using this technique makes gene replacement and editing possible. For example, expression patterns can be studied as it allows the insertion of in-frame fluorescent proteins. Alleles engineered in this way should retain the expression properties of endogenous genes. Thus, this approach circumvents many problems associated with constructs placed within extrachromosomal arrays, such as overexpression, copy-number–generated gene silencing, or defective expression in germ line. The latter in particular is a major problem associated with extrachromosomal arrays in *C. elegans*; see below. Together with availability of databases and libraries containing large number of strains with localised *Mos1* insertions (see above), this provides a powerful approach to manipulating many *C. elegans* genes. More recently, Frokjaer-Jensen and coworkers developed a new version of gene targeting using *Mos1* mobilisation followed by repair for the production of deletions, i.e., *Mos*DEL (*Mos1*-mediated deletion)[46]. This technique allows the deletion of up to 25 kb of DNA and also permits the insertion of a selection marker to follow the targeting events. *Mos1* insertion sites are now known within 25 kb of most genes in *C. elegans*, thus, in principle, allowing the targeting of nearly any gene.

Although not a gene-targeting method, it is also useful to mention a method and toolkit developed by the same authors to insert transgenes in the genome in predefined loci using a similar strategy, so-called *Mos*SCI (Mos1-mediated single copy transgene insertion)[47]. This method permits the production of transgenic strains carrying different constructs inserted in the same locus[47]. In combination with the availability of deletion mutants, for many genes it provides an adaptable approach to gene substitution within worms.

## Modification of the Genome by Zinc-Finger Nucleases

Zinc-finger nucleases are engineered chimearic molecules that consist of Cys2His2 zinc-finger DNA binding domains attached to a peptide with nonspecific nuclease activity derived from the restriction enzyme *Fok*I[48]. The specific binding properties of the protein depend on the exact nature of the zinc-finger domains that it contains. Thus, zinc-finger nucleases can bind to specific DNA sequences and induce DSB (see Porteus and Carroll[49] for a review). A single zinc-finger domain can make contact with a single DNA triplet[50]. The key to using this ability to achieve targeted DNA binding has been the identification of naturally occurring, or artificiality produced, zinc-finger domains with a wide range of specificities. Zinc-finger domains are now available that bind to the majority of the 64 possible DNA triplets (see Urnov et al.[51] for an update). Therefore, by concatenating three or more different zinc-finger domains, it is possible to produce a zinc-finger–containing molecule that will specifically bind to a particular sequence (Fig. 3). Moreover, because the *Fok*I nuclease domains autoassemble as a dimer, two sets of zinc-fingers will be required to bind DNA (Fig. 3), hence augmenting the specificity of binding. However, generating functional constructs has been a bottleneck in the use of zinc-finger nucleases. To overcome this, a range of approaches to producing new specificities has been developed (see de Souza[52] and references therein) and banks of zinc-finger nucleases are being generated[53,54]. In addition, expression of a specific zinc-finger nuclease will result in the production of a DSB in the target DNA. This can be exploited to produce a variety of possible outcomes (see Urnov et al.[51]), including mutagenic changes at the cleavage site (Fig. 3). A full strategy for the design of zinc-finger nucleases to target *C. elegans* genes is described by Carroll et al.[55].

Zinc-finger nucleases have been used to modify the *C. elegans* genome (Table 1)[56]. Morton and coworkers designed a zinc-finger nuclease, which recognises six GNN triplets, that is 5′-(NNC)3N6(GNN)3-3′ when assembled as a dimer (Fig. 3). They then searched amongst the potential targeting sites in the worm genome for those that contained a restriction site flanked by the recognition sites for the zinc-finger construct, thus providing easy identification of the targeted DNA[56]. A suitable site was identified on the X chromosome. Transgenic animals carrying the zinc-finger nuclease construct under the control of the heat shock promoter were then produced. After induction of the nuclease, a range of different events in the site (i.e., deletions, insertions, and combined) were observed, demonstrating the feasibility of this approach (Table 1). However, the changes shown by the authors were not inheritable, suggesting that the zinc-finger nucleases were either not expressed or not functional in the germ line. The explanation is probably, at least at the first level, the former, i.e., that there was no expression from the transgene in the germ line. This is a recurring theme in attempts to exploit gene-modifying enzymes provided on transgenes in *C. elegans.*

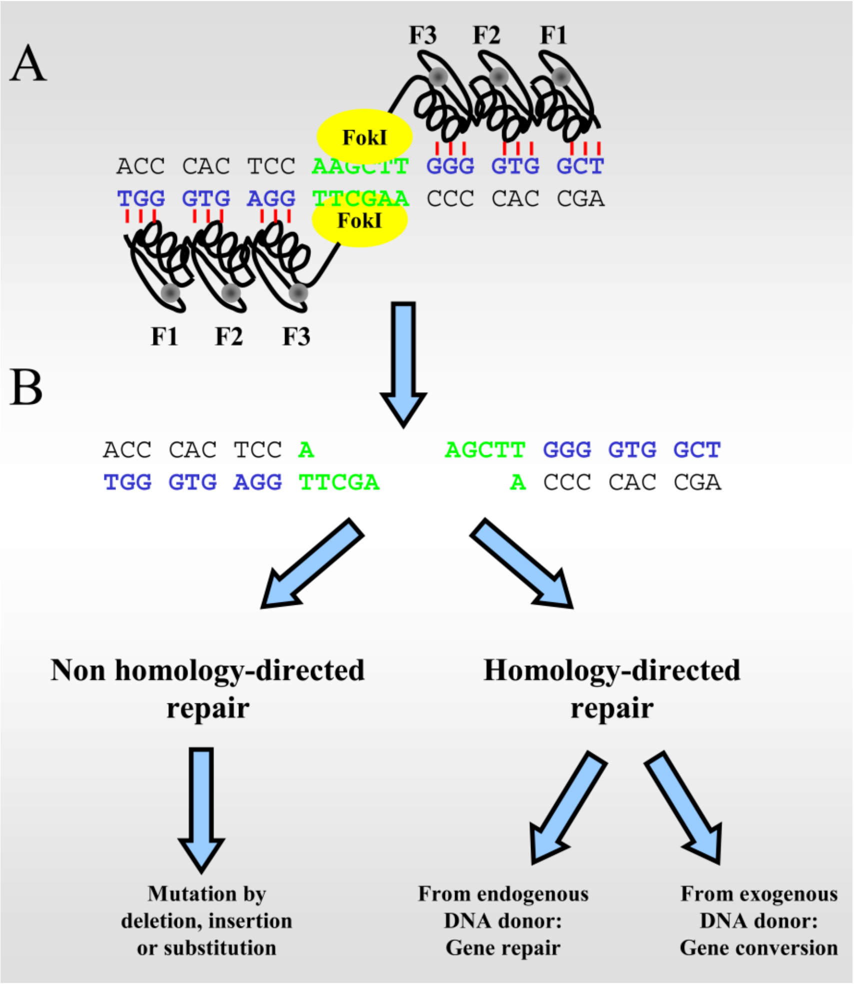

**FIGURE 3.** Mutagenesis induced by zinc-finger nuclease activity. (A) Cartoon of the structure of the zinc-finger nuclease dimer described by Morton et al.[56] containing three fingers (F1, F2, and F3). Black lines represent the amino acid sequence of the peptide. Grey dots represent zinc ions. Red bars represent the binding of each finger to the triplet motif specifically recognised by the finger. The *Fok*I nuclease domain is labelled yellow. (B) Digestion of the target by the zinc-finger nuclease induces DSB. This lesion can be repaired by end-joining ligation, which may induce mutagenesis of the *locus*. Otherwise the lesion can be resected by nucleases and hence the gap will be repaired by copying the sequence from a homologous sequence resulting in gene repair or gene conversion (see legend to Fig. 1).

The bipartite structure of the DNA binding domain and *Fok* nuclease has also been exploited in TALENs (transcription activator-like effector nuclease), which utilise the extraordinary DNA binding abilities of TAL elements. There are, as yet, no reports of the use of such molecules in *C. elegans*, but they offer significant promise in overcoming some of the difficulties associated with developing effective zinc-finger domains (see Cermak et al.[57] and Li et al.[58] and references therein).

## Early Attempts at Gene Targeting by Homologous Recombination in *C. elegans*

Gene targeting aims to modify genes by introducing exogenous DNA that contains sequences homologous to the target DNA. The homologous DNA sections (arms) flank nonhomologous DNA or a deletion. Homologous recombination results in the exogenous DNA being incorporated into the target, thus modifying the gene. This strategy has been used extensively and systematically in yeast and mouse for many years (see Muller[59] and Sorrell and Kolb[60] for reviews), and has had an enormous impact on, for example, studies of mammalian biology[61] and biomedicine[62,63,64,65]. Further, the use of gene targeting together with the recombinases Flp and Cre allows the production of conditional knock-outs, a particularly important tool in the studies of those disease-related genes in which complete loss of function is incompatible with life (reviewed by Branda and Dymecki[66]). Despite their inherent power as an enabling technology, such strategies have not been developed in *C. elegans*, until recently.

Transgenes are usually introduced into *C. elegans* by injecting DNA into the cytoplasm of the syncytial part of the worm gonad[67]. Generally, this technique results in the creation of DNA molecules formed from multiple copies of the injected DNA. These "extrachromosomal arrays" are then inherited in a non-Mendelian way[67]. One disadvantage of such arrays is that the expression of transgenic constructs from extrachromosomal arrays may not reproduce that of the endogenous genes; indeed, as noted above, most such transgenes are not expressed in the germ line. Broverman and coworkers therefore decided to use a different strategy to study gene expression of vitellogenin genes. Their aim was to produce chromosome-integrated transgenes[68]. To do so, they injected DNA directly into single meiotic oocytes[69] as opposed to the syncytial part of the gonad. In the course of this work, the authors serendipitously obtained the first ever–reported event of gene targeting in *C. elegans*, when the injected *vit-2* construct targeted the *vit-2* gene itself (Table 1)[68]. This targeting event appeared to occur through a single recombination event, between the injected supercoiled plasmid and the *vit-2* locus, and resulted in the insertion of the construct[68]. Although the *vit-2* locus was successfully targeted, the microinjection of oocytes is so laborious and the efficiency of homologous integration so low (~3% of the integrated lines)[68], this strategy has not been employed to produce other gene knock-outs.

## Gene Targeting by Biolistic Transformation

Low frequencies of homologous recombination during gene targeting occur in many organisms, but do not prevent the use of gene targeting. Although little can be done to overcome natural limitations in homologous recombination frequencies, there are other ways to make such strategies more readily usable. Berezikov et al. set out to improve the application of gene targeting by implementing a standardised method based on biolistic transformation[70]. Biolistic transformation had previously been shown to be effective in *C. elegans*[71,72]. Praitis and coworkers used gold beads coated with the transforming DNA and a Bio-Rad Biolistic PDS-1000/He apparatus to bombard worms. They made an important discovery: that when introducing DNA by this technique, low-copy number insertions, within the genome of *C. elegans*, were created at much higher frequency than when using standard microinjection protocols[71]. In these experiments, between 0.3 and 1.2 transformants per bombardment were obtained. These transformants included both chromosomal insertions and extrachromosomal arrays[71]. The frequency of transformants in which the transgene was inserted into the genome ranged from 13 to 35%[71]. Berezikov and colleagues argued that scaling up this technique might result in enough insertional events to make it probable that rarer targeted homologous events could be detected[70]. To increase the number of transformants, the authors also used the Bio-Rad PDS-1000/He device, but with a Hepta adaptor (Bio-Rad), which allows the exposure of a 9-cm Petri dish covered with worms, instead of the 2-cm Petri dish used by Praitis et al. This increased the number of animals bombarded from ~75,000[71] to ~350,000[70]. To provide the large number of animals required, they used culture plates seeded with bacteria and chicken egg[70,73]. To further improve the technique, the authors linearised constructs prior to bombardment[70] as free DNA ends emulate DSB and thus induce homologous recombination[36,37,38]. Using this approach, they were able to obtain 4.6 to

14.3 transformants per bombardment[70] compared to the previous figure of 0.3 to 1.2[71], hence achieving the goal of scaling up the technique. Amongst the products, they observed that an average of about 30% contained insertional events, a figure which is comparable to previous work[70].

The construction of appropriate targeting vectors is an important part of successful gene-targeting protocols. As a selectable marker for worms carrying transgenic DNA, Berezikov and colleagues used the *unc-119* gene. *unc-119(ed3)* mutant worms have extremely uncoordinated locomotion and an inability to produce a stress-resistant larval stage, the so-called Dauer larva. Dauer larvae can survive starvation for several months, whereas other life cycle stages perish. Introduction of a wild-type *unc-119* transgene is sufficient to rescue these phenotypes so that worms move normally and are able to survive starvation. Hence, after bombardment, animals were left on plates to starve and worms that showed wild-type locomotion were isolated as potential transformants[70]. In addition to this positive selection marker, the authors targeted genes with readily detectable phenotypes, such as *unc-22* and *unc-54*, which themselves show strong uncoordinated locomotion when disrupted[70], hence making it possible to identify potential targeting events based on phenotype. By using all of these improvements, Berezikov and colleagues obtained four *unc-22* knock-outs and one *unc-54* knock-out (Table 1). Jantsch and colleagues used a similar strategy to target the *zhp-3* gene[74]. In this case, they used the predicted embryonic lethal phenotype of *zhp-3* as an additional selection tool. The proportion of targeted insertions by homologues recombination amongst the total transformant population in these experiments was 0.43, 0.36, and ~1% for *unc-22, unc-54,* and *zhp-3,* respectively. This suggests that roughly 1 to 3% of integration events result from homologous recombination, although no independent estimate for the total number of integration events was made in the Jantsch experiments. These results clearly demonstrate that gene targeting is feasible in *C. elegans*, however the frequencies involved mean that large numbers (typically ≥25) of bombardments are required to generate each targeting event.

## Improved Gene Targeting by Biolistic Transformation using Counter Selection

The experiments described above clearly demonstrate that gene targeting can be used in worms. However, in all three cases, genes with a clear, predicted phenotype were targeted, allowing some selection by phenotype for likely gene-targeting events amongst the transformants. A more likely scenario is that a researcher wishes to generate a gene knock-out in a gene for which the phenotype is unknown and which may be relatively subtle; for example, changes in behaviour. As discussed above, gene targeting has the advantage of generating a precise and clean modification. This can be useful in some circumstances; for example, in analysis of the *plc-4* gene[75]. As part of our work to dissect $IP_3$ (inositol 1,4,5-trisphosphate) signalling in *C. elegans* (e.g., Vazquez-Manrique et al.[76] and Walker et al.[77]), we wished to analyse the role of PLC-4, the worm homologue of phospholipase C-δ, the function of which is poorly understood. RNAi of *plc-4* gave no apparent phenotype, a result that could be a true reflection of knock-down or may reflect a lack of knock-down in the whole animal or appropriate tissues. Molecular analysis of transcripts from an existing deletion allele suggested that some transcripts would encode in-frame mRNAs that could produce truncated, but potentially functional, proteins (Vazquez-Manrique and Baylis, unpublished data). Hence, we sought to ablate *plc-4* by gene targeting using a method derived from the protocol by Berezikov and colleagues.

In order to facilitate the identification of homologous integration events amongst the transformants, we employed both a positive selection marker and a counter selection marker. The counter selection marker is placed outside of the homology arms and will therefore be lost in events mediated by homologous recombination, but is likely to be retained in both random integration events and nonintegrated extrachromosomal arrays (Figs. 4 and 5). Thus, the counter selection marker provides a mechanism for detecting probable targeting events amongst the transformants (Fig. 5). As a positive marker, we used the so-called *unc-119* "minigene" (from now on referred to as *unc-119p::unc-119*) (Fig. 4)[78]. Using a smaller version of this marker facilitates cloning of larger homology arms in the targeting vector, which in turn may increase the probability of homologous recombination events. As a counter selection

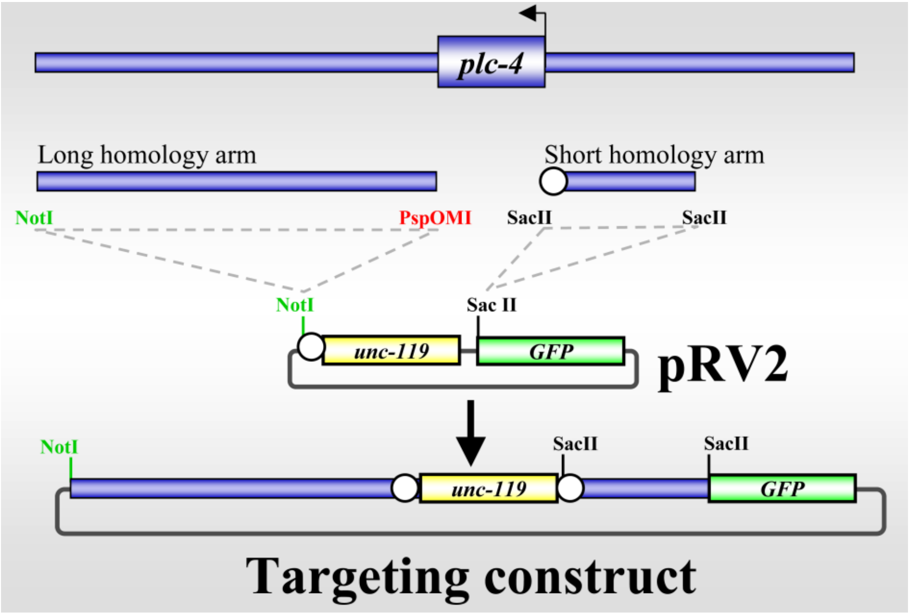

**FIGURE 4.** A dual-selection vector for gene targeting in *C. elegans*. pRV2 contains two selection markers, *unc-119p::unc-119* (yellow box) and *myo-2p::GFP* (green box) in pGEM-5Z. Flanking the left site of *unc-119p::unc-119,* there is an FRT sequence and a *Not*I restriction site. Between both markers, a *Sac*II restriction site is present. To produce a targeting vector, a long homology sequence, corresponding to the region downstream of the stop codon of the target gene, is introduced into the *Not*I site. A short arm, homologous to the sequence upstream of the start codon of *plc-4*, is amplified by PCR together with an FRT site provided in the left primer and cloned within the *Sac*II site. Blue bars show genomic DNA sequences homologous to the region flanking the gene to be targeted. The *plc-4* locus is shown at the top of the figure (not to scale). Empty circles represent FRT sequences. The thin grey bar represents the pGEM-5Z backbone.

marker, we used *myo-2p::GFP* (Fig. 4). *myo-2p::GFP* expresses GFP in the pharynx of the worms, which is readily scored under a dissecting microscope equipped with fluorescence. Both markers were cloned in pGEM-5Z (Promega) to produce a vector (pRV2 [Fig. 4]) that can, in principle, be used to target any gene (see Fig. 4 for explanation). To target *plc-4*, we inserted asymmetric homology arms, a long (6 kb) homology arm and a short homology arm (2.2 kb) (Fig. 4). The construct was linearised prior to bombardment to further induce homologous recombination. Finally, we included FRT sites (i.e., the substrate of Flp recombinase), flanking the *unc-119p::unc-119* cassette (Fig. 4). This would facilitate the removal of the selection marker using Flp recombinase in order to obtain a clean knock-out.

We performed 50 bombardments on *unc-119(ed3)* worms, producing 230 transgenic strains, i.e., 4.6 strains per shot[75], a figure similar to that obtained by Berezikov and colleagues[70]. After visual examination of the resulting transformants, under the dissecting microscope, we isolated 28 strains that did not show GFP expression. After PCR analysis of these animals, we found one mutant allele of *plc-4*, named *jw1*[75]. The proportion of targeting events among all transformants was again low, 0.43%, although comparable to those obtained by Berezikov et al.[70]. However, as we used the counter selection marker *myo-2p::GFP*, we had to screen only 28 strains, showing that this strategy reduces the effort required in screening for targeting events.

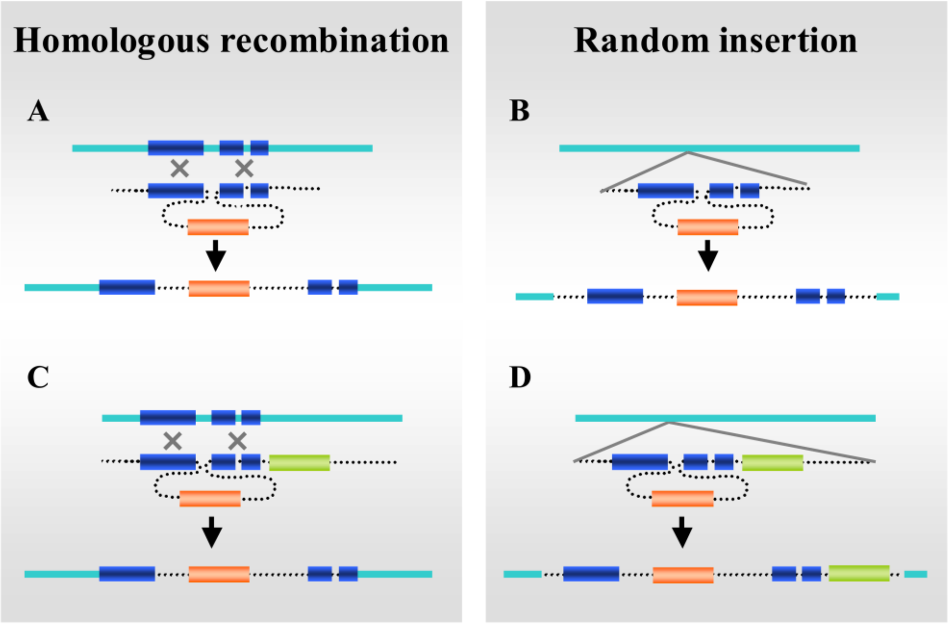

**FIGURE 5.** Gene targeting by biolistic transformation using counter selection. A and B show the result of a homologous recombination targeting event and a random integration in an unspecific locus of the genome, using a targeting vector containing just one selection marker. Both events are undistinguishable. C and D show the same events when a vector containing two selection markers, such as our targeting construct, is used. In this case, it is easy to discriminate probable homologous recombination events from nonspecific integration events.

## Attempts to Exploit Flp-Mediated Recombination

As stated above, we introduced FRT sequences flanking the selection marker *unc-119p::unc-119*. Flp recombinase has been demonstrated to function in somatic cells in *C. elegans*[79,80]. To remove the marker, we simultaneously introduced constructs carrying Flp recombinase under the control of the heat shock promoters *hsp-16-2p* and *hsp-16-41p*[81] into the *plc-4(jw1)*; *unc-119(ed3)* animals. We induced the expression of Flp recombinase by heat shock in adult worms and observed that their offspring showed strong uncoordination, suggesting that the *unc-119p::unc-119* cassette had been lost due to recombination. Thus, Flp-mediated recombination can be used to remove selection markers or, presumably, make other changes in targeted genes. However, this change was not heritable, suggesting that it had occurred in somatic cells. In order to create a clean knock-out, Flp-mediated excision in the germ line is necessary. Despite extensive attempts using a number of strategies, we were unable to achieve a result that is in agreement with other studies[79,80]. Once again, the difficulty of obtaining gene expression from transgenes in the germ line is a significant problem. Nevertheless, excision in somatic cells has significant potential as a tool for conditional mutant generation. We therefore tested for tissue-specific excision in the nervous system and in body wall muscles with some success. These experiments and those of others suggest that conditional knock-out is feasible in *C. elegans*.

## DISCUSSION

The ability to perform reverse genetics is central to programs to, for example, study the role of human disease gene orthologues, dissect the roles of genes identified in high throughput and systematic approaches, or to integrate biochemical and *in vivo* function. It is desirable not only to be able to ablate gene function, but also to be able to modify genes to produce defined alterations in proteins within their endogenous genomic environment. The *C. elegans* community has developed a substantial toolkit to manipulate the *C. elegans* genome. These approaches are discussed above; alternative approaches used in other organisms, such as gene editing by oligonucleotides, have been attempted in *C. elegans*[82], but without success.

To date, the simplest and most productive methods to produce gene knock-outs have employed random mutagenesis by chemicals, ionising radiation, or transposon mobilisation. These techniques require extensive investment in the screens required to identify the desired genomic lesion. Random mutagenesis usually produces more than one lesion per genome, which requires strains to be cleaned up by outcrossing. In extreme cases, secondary lesions may be located too close to the desired mutation to be removed with ease. To avoid the disadvantages associated with random mutagenesis, it is highly appealing to use controlled methods to target genes. In *C. elegans,* the only methods described, so far, that allow the introduction of predefined changes in the genome are gene conversion and gene targeting by biolistic transformation.

Gene conversion can be achieved by mobilisation of transposable elements, which induce a lesion in the desired target gene. This lesion may be repaired by endogenous cellular protective mechanisms. If this repair occurs in the presence of transgenic constructs, exogenous DNA may be used as a template and hence controlled changes may be introduced in the gene. This strategy to alter the genome is extremely powerful since it allows the production of knock-out and knock-in events. Mobilisation of endogenous transposons, such as *Tc1*, may induce undesirable mutations. Hence, the best option to induce controlled changes in the genome using transposable elements is the use of MosTIC, mediated by the exogenous element Mos1[45]. One hurdle to the use of *Mos1* is the requirement for the presence of a transposon insertion within 0.5 kb of the target region[45]. Therefore, many *loci* are currently excluded and it may be hard to identify suitable insertions for some loci. As discussed above, programs to produce *Mos1* insertions in every *loci* of the genome[33,83] are increasing the number of readily targeted *loci*. The use of zinc-finger nucleases has proven to be useful to target a genomic locus in *C. elegans*, and shows potential to be used for gene conversion purposes. Improvements in the production of these and similar nucleases will greatly improve the utility of this approach. However, the use of these enzymes is, to date, restricted to somatic cells in *C. elegans*[55]. Here and elsewhere (e.g., the use of Flp recombinase), difficulties associated with the germ line expression of transgenes are limiting the facile use of such approaches in *C. elegans*.

Gene targeting by biolistic transformation is an alternative to gene conversion, to produce controlled changes in the genome. This strategy has the advantage over other methods of gene manipulation in that it allows, in theory, the targeting of any *loci* of the genome. It should be noted, however, that gene targeting by homologous recombination shows a position effect in mammalian systems, including human cells[84], and this may also apply to the genomes of other organisms, including *C. elegans*. Gene targeting by homologous recombination allows both gene knock-out and knock-in. The use of Flp recombinase to produce conditional gene knock-out in somatic cells further increases the value of this strategy. However, relatively few genes have been targeted using these approaches thus far (Table 1). This reflects the fact that the technique still remains very labour intensive. In this regard, it would be desirable to perform systematic studies to optimise the conditions of gene targeting to further increase the efficiency. The use of improved positive selection, such as antibiotic selection methods for example[85,86], might also help to further develop gene targeting. The most useful improvement would be a method to increase the level of homologous recombination or a significantly more efficient method of producing transformants in large numbers.

A wide range of approaches exist to manipulate the *C. elegans* genome. The advent of next-generation sequencing and deep sequencing strategies for exploiting random mutagenesis and forward genetic screens is having an immense impact on closing the genotype-phenotype gap. As the depth in which we need to dissect the link between genes, proteins, function, and whole animal biology increases, there will also be an increased emphasis on an ability to make a range of carefully designed and relatively subtle changes in the gene of interest. New and improved methods are being developed, including those that enable controlled changes to be made in the genome. Many of the approaches we have described are supported by resources that are made available to the whole community. Together, these methods and resources enable reverse genetic strategies to be applied to nearly any gene of interest, be that the homologue of a disease-related gene, an orphan gene identified in genomic or postgenomic studies, or a gene with poorly characterised whole-animal function.

## ACKNOWLEDGEMENTS

This work was supported by the BBSRC (RVM) and MRC (HAB). HAB was an MRC Senior Fellow.
